# Spatio-temporal variations in water quality of a river–lake system during restoration treatments

**DOI:** 10.1007/s10661-022-10307-1

**Published:** 2022-08-04

**Authors:** Joanna Rosińska, Michał Rybak, Katarzyna Kowalczewska-Madura, Renata Dondajewska-Pielka, Anna Kozak, Ryszard Gołdyn

**Affiliations:** 1 Department of Environmental Medicine, Poznan University of Medical Sciences, Rokietnicka 8, 60-806 Poznań, Poland; 2 Department of Water Protection, Faculty of Biology, Adam Mickiewicz University, Uniwersytetu Poznańskiego 6, 61-614 Poznań, Poland

**Keywords:** Flow-through lake, Limited restoration, Outflow, Shallow lake, Sustainable restoration, Tributary

## Abstract

To fill the knowledge gap about the functioning of the lake–river system subjected to restoration treatments, two tributaries, a shallow, restored lake and its outflow, were examined. The quality of water inflows, lake and outflow was compared before (BR), during sustainable (SR, deep water aeration, phosphorus inactivation and biomanipulation for 3 years) and limited lake restoration (LR, only aeration for 2 years). Physico-chemical parameters were analysed monthly at five stations. The nutrient concentrations at the inflows decreased over the years due to the improvement of water and sewage management in the catchment (in Mielcuch from 18.0 to 8.0 mgN L^−1^ and 1.0 to 0.6 mgP L^−1^). The decline at the outflow was the result of a better quality of water at the tributaries and SR in the lake. During LR, decrease of phosphorus concentration still occurred (0.11 mgP L^−1^), but nitrogen concentration slightly increased (3.9 mgN L^−1^). Although the outflowing waters still transported a high content of chlorophyll *a* and suspended solids during SR, their amount was lower (34.5 μg L^−1^ and 17 mg L^−1^, respectively) than that during BR and LR. During restoration, it is significant to monitor the water quality not only in the lake but also at the outflow. The slow deterioration of water quality at the outflow indicated that introducing changes in the applied restoration methods must be done carefully because the previously achieved effect may be lost. Hence, restoration of the upstream lake and good quality of its tributaries are of great importance for water bodies located downstream.

## Introduction

The improvement of lake water quality under the European Union Water Framework (Directive, 2000) is challenging, especially in flow-through lakes, where it is impossible to eliminate all sources of pollution (Dunalska et al., 2018; Tekile et al., 2015). The river and the lake form an integral whole in the river–lake system, and they have a significant impact on each other. Rivers flowing through lakes influence their water balance and chemical parameters (Klimaszyk et al., 2015; O’Hare et al., 2018; Wetzel, 2001). They also impact the biotic conditions in the lake ecosystem (Krepski & Czerniawski, 2018). While lakes, usually shallow, non-stratified and eutrophic, being common elements of lowland river systems, disturb the natural river continuum by altering the hydrological, physico-chemical, and biological parameters of water (Hillbricht-Ilkowska, 1999; Ward & Stanford, 1983; Wetzel, 2001) and extending the pollution cycle (Kufel & Kufel, 1997). It is well known that as a result of a sudden slowdown of the river flow into the lake, mineralisation of organic matter occurs, with the deposition of delivered and newly created suspensions to the bottom sediments (Kufel, 1993; Pytka et al., 2013). Thus, lakes become a typical sink of pollutants (Hatvani et al., 2017; Pytka et al., 2013; Tian et al., 2017), and downstream water bodies may be less polluted with nutrients and organic matter (Gołdyn & Szeląg-Wasielewska, 2004; Stanford et al., 1988). However, over time, flow-through lakes become overloaded with nutrients and transform into a source of pollution for the river waters below. The transported material is only processed and exported further, which in turn causes the outflow water to be additionally enriched with organic matter produced in the lake (Hillbricht-Ilkowska, 1999; Teodoru & Wehrli, 2005). This poses a threat not only for the river but also for the downstream lakes. Thus, restoration treatment is needed to improve water quality in the lake, which also affects the river flowing out of the lake. It may have different effects for ecological functioning (biodiversity; quality of river and lake ecosystems below lake with poor/bad state, etc.) and societal value of water ecosystems (reservoir for drinking water, water retention, fishing, recreation) (Ekvall et al., 2014).

Although processes occurring in the river–lake system are well-known, there is still little knowledge about changes in such systems when the lake is restored. This information is extremely important as more and more flow-through lakes are currently undergoing restoration (Dondajewska et al., 2020).

The aim of the study is to compare the water quality of two tributaries flowing through a shallow urban lake that was subjected to the restoration processes, the lake and its outflow. Data were collected before, during restoration treatments (with three applied methods) and under the limited restoration (with one applied method). The observation of water parameters at the inflows, two lake stations and outflow allowed an analysis of the temporal and spatial changes of water quality during the restoration activities applied in the lake. It is hypothesised that (1) restoration activities carried out on the flow-through lake will contribute to the improvement of the water quality, resulting in lower nutrient and chl *a* concentrations at the outflow, and (2) limiting restoration activities to one method will result in a worse quality of outflowing water than during the period of more intensive restoration treatments (with three methods).

## Materials and methods

### Study area

**Fig. 1:**
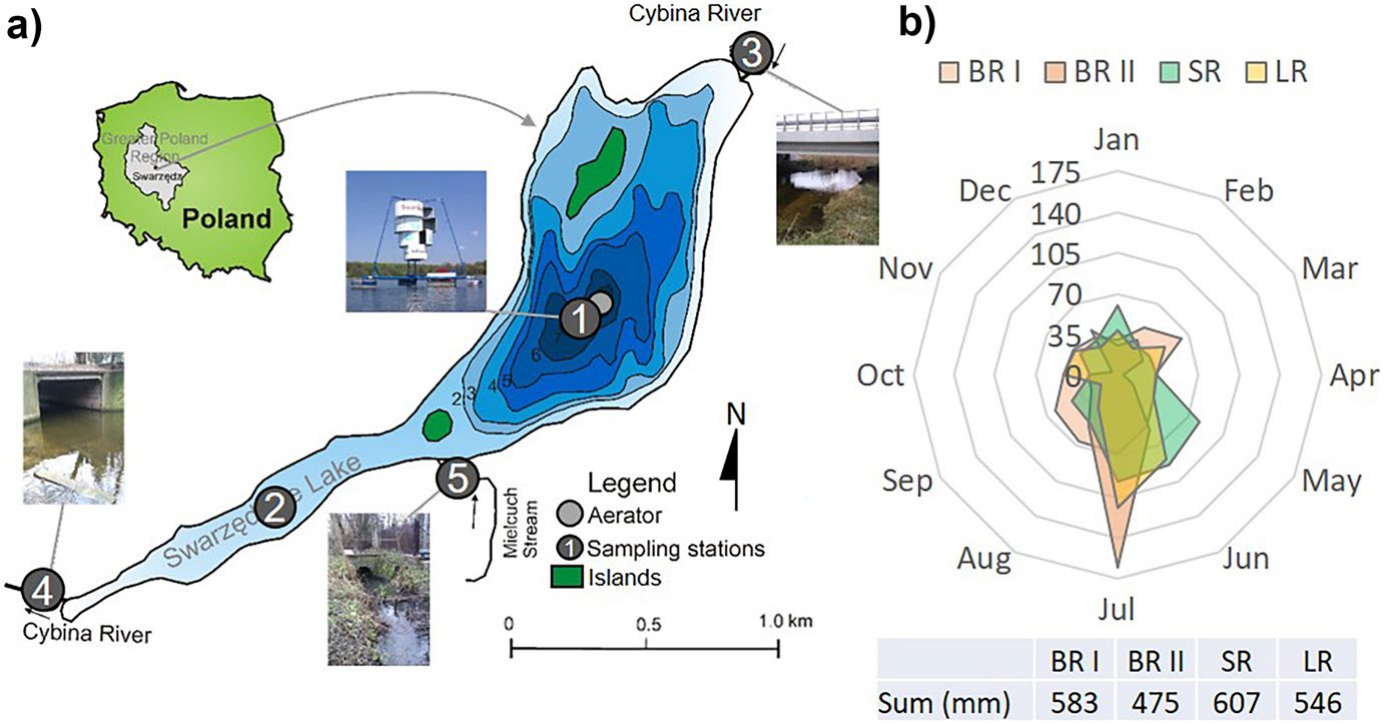
**a** Sampling stations: 1, the deepest part of the lake (near aerator); 2, shallower part of the lake; 3, Cybina River Inflow; 4, Cybina River Outflow; 5, Mielcuch Stream (Kozak et al., 2018, changed) and **b** precipitation in Poznań before (BR I, BR II), during sustainable (SR), and limited restoration (LR).

The analysed system consists of two tributaries, lake and outflow (Fig. 1a). The main tributary is the Cybina River, and the second, smaller tributary is the Mielcuch Stream (Fig. 1a). Swarzędzkie Lake (52°24′49″N, 17°03′54″E) is a shallow, flow-through urban lake (depth 7.2 m, surface 93.7 ha). It has been characterised by hypertrophy and strong cyanobacterial blooms (mainly *Pseudanabaena limnetica* and *Aphanizomenon gracile*), low Secchi depth and a lack of submerged macrophytes for more than 10 years because Swarzędzkie Lake was a sewage receiver until 1991 (Kowalczewska-Madura & Gołdyn, 2006; Kozak et al., 2014; Rosińska & Gołdyn, 2015). Thus, Swarzędzkie Lake could not be used for recreational purposes. The mean water retention time is 37 days (Szyper et al., 1994). The tributaries of the Swarzędzkie Lake are rich in nutrients that accumulate in lake sediments (Barałkiewicz et al., 2008; Kowalczewska-Madura & Gołdyn, 2006; Szyper et al., 1994).

The Cybina River is a lowland river about 42.0 km long, which flows to the Warta River in Poznań, its right-bank tributary. The area of its basin, up to the outflow from Swarzędzkie Lake, covers 17,826 ha. About 75.5% is occupied by agricultural fields, 18.0% by forests, and over 5.0% by buildings. There are numerous fish ponds in the river valley (total area of 144 ha, volume 2·10^6^ m^3^), natural lakes and artificial reservoirs of high trophic state (Szyper et al., 1994). An additional periodic source of nutrients is the autumn inflow of water drained from fish ponds that carry high loads of organic matter, nitrogen and phosphorus (Kowalczewska-Madura et al., 2005; Szyper et al., 1994).

The Mielcuch Stream, the second, smaller tributary of the lake, is more polluted by nutrients (even 20.6 mg N L^−1^, 1.8 mg P L^−1^) and industrial contamination (from car wash and roads), as it is the main receiver of rainwater and illegal sewage discharges (Gołdyn & Grabia, 1998). It is quite short, 4.7 km, and the area of its basin is only 624 ha (Szyper et al., 1994), mainly urbanised (60.5%) and agricultural (38.5%) (Gołdyn & Grabia, 1998). The water flow in this stream is about tenfold less than that in the Cybina River. The Mielcuch Stream flowing through agricultural areas is an open ditch, while in the city of Swarzędz, it is converted into a rainwater collector, and at its mouth, it once again becomes an open ditch.

### Different treatment periods

To improve water quality, eliminate cyanobacterial water blooms (in 2011: cyanobacterial bloom was noted from June to November, the lowest water transparency was 0.5 m, the highest chlorophyll *a* concentration 278 μg L^−1^ (Kozak et al., 2014)) and restore the ecosystem services of the Swarzędzkie Lake for recreational use, sustainable restoration was applied (Table 1). This means that bottom-up and top-down treatments were conducted in the lake simultaneously at low intensity to initiate natural processes for decreasing the nutrient concentrations and reconstructing the composition of the aquatic organisms (Gołdyn et al., 2014; Rosińska et al., 2018). Three different methods: phosphorus inactivation using low doses of iron sulphate and magnesium chloride (a few kilogrammes per hectare), aeration of waters above the bottom sediments using a wind-driven aerator, biomanipulation with cyprinid catches and restockings with pike and zander fry were applied from autumn 2011 until December 2014 (Table 1; for more details, see Rosińska et al., 2018, 2019). However, due to a lack of funding, since 2015, restoration has been limited to one method, i.e. deep water aeration (Kowalczewska-Madura et al., 2020; Kozak et al., 2018).

**Table 1:** Details of restoration treatments in Swarzędzkie Lake (Kowalczewska-Madura et al., 2020; Rosińska et al., 2018, 2019)

Name of period	Time	Applied treatments
	Autumn 2011	Fish removal (700 kg)—mainly roach, white bream and crucian carp
SR, sustainable restoration	2012	Water aeration by pulverising aerator Phosphorus inactivation (9 times) Biomanipulation (70 kg autumn pike fry)
	2013	Water aeration by pulverising aerator Phosphorus inactivation (5 times) Biomanipulation (70 kg)
	2014	Water aeration by pulverising aerator Phosphorus inactivation (5 times) Biomanipulation (7200 psc. of summer fry and 200 kg of autumn fry)
LR, limited restoration	2015–2016	Water aeration by pulverising aerator

### Field data, sampling and laboratory analyses

Precipitation data were obtained from the nearest meteorological station in Poznań ca. 12 km from Swarzędzkie Lake (for the period 2000–2002 from the Report (2003) and 2011–2016 from the Institute of Meteorology and Water Management (https://klimat.imgw.pl/pl)).

The research was carried out at five sampling stations (Fig. 1a). Two stations were located in the lake—at the deepest part of the lake, near the aerator (Station 1) and in the middle of the shallower part of the lake (Station 2). Also, three stations were located at the rivers: the inflow to Swarzędzkie Lake (Cybina Inflow, station 3), the outflow from Swarzędzkie Lake (Cybina Outflow, station 4) and near the mouth of the Mielcuch Stream to Swarzędzkie Lake (Mielcuch, station 5) (Fig. 1a). Samples were collected in three periods: before restoration (BR I, 2000–2002; and BR II, 2011), during sustainable restoration (SR, 2012–2014) and limited restoration (LR, 2015–2016). The BR period was divided into two time intervals because water quality during the BR II period was extremely bad and differed from the BR I period. Water for physical and chemical analyses was sampled monthly from the middle river current 0.15 m below the surface (total sample size *n* = 282; 94 samples for each station) and stations located in the lake from the surface layer (mean from 0.2 m and 1.0 m depth; total sample size *n* = 295; 185 samples at Station 1 and 110 samples at Station 2). Samples at Station 2 were collected only during the restoration periods.

Temperature, pH, conductivity and dissolved oxygen content were measured in the field with a YSI Pro Plus multiparameter meter. Based on the chemical analyses carried out in accordance with Polish Standards (Elbanowska et al., 1999), the concentrations of soluble reactive phosphorus (SRP) and total phosphorus (TP), as well as ammonium nitrogen (N-NH_4_), nitrate nitrogen (N-NO_3_), nitrite nitrogen (N-NO_2_) and total nitrogen (TN), were determined. The concentration of chlorophyll *a* (chl *a*, spectrophotometrically after filtration on GF/C and 90% acetone extraction) and the content of total suspended solids (TSS) were also measured. Data from 2000 to 2002 were published by Kowalczewska-Madura (2003; 2005) and were used here to support BR data. Some of the data, detailed analyses of water quality changes in Swarzędzkie Lake, were published by Kozak et al. (2014), Rosińska et al. (2018) and Kowalczewska-Madura et al. (2020).

### Statistical analysis

The Shapiro–Wilk test was used to evaluate the normal distribution of the results, while the Levene test was applied to assess the equality of variances for groups. Not all data was characterised by a normal distribution. A comparison of atmospheric precipitation in the studied periods was made using the Kruskal–Wallis test (*n* = 108). The two-way ANOVA with post hoc HSD Tukey was applied to find any spatio-temporal changes in the analysed parameters. Spearman’s correlation (Spearman’s *ρ*) between nutrients and periods at the outflow was done. The canonical variates analysis (CVA) was used to analyse which environmental variables were the most important in differentiation of the analysed watercourses. It was also employed to assess how inflows and the lake affected the outflow, how the water quality at the outflow changed during the studied periods and which parameters were the most important in each watercourse. The outputs have not been transformed. A global Monte Carlo test was performed to determine the significance of the correlations in CANOCO. The significance threshold in all statistical analyses was *p* < 0.05. Statistical procedures were calculated using Statistica 13.1 software and Canoco for Windows 4.5 software package (Ter Braak & Šmilauer, 2002).

## Results

### Precipitation

The mean sum of annual precipitation was typical for this region during the studied periods and ranged from 475 mm (BR II) to 607 mm (SR; Fig. 1b). The annual rainfall in 2011 and 2015 was lower than that in the multi-year period for Poznań (520 mm), i.e. 475 mm and 438 mm, respectively (dry years). Higher annual precipitations were noted during the SR and LR periods (the wettest was 2012, 669 mm; and 2016, 655 mm). The rainiest months were June and July, which is typical for the temperate climate zone (Fig. 1b). Differences between periods were not statistically significant.

### Water quality

**Fig. 2:**
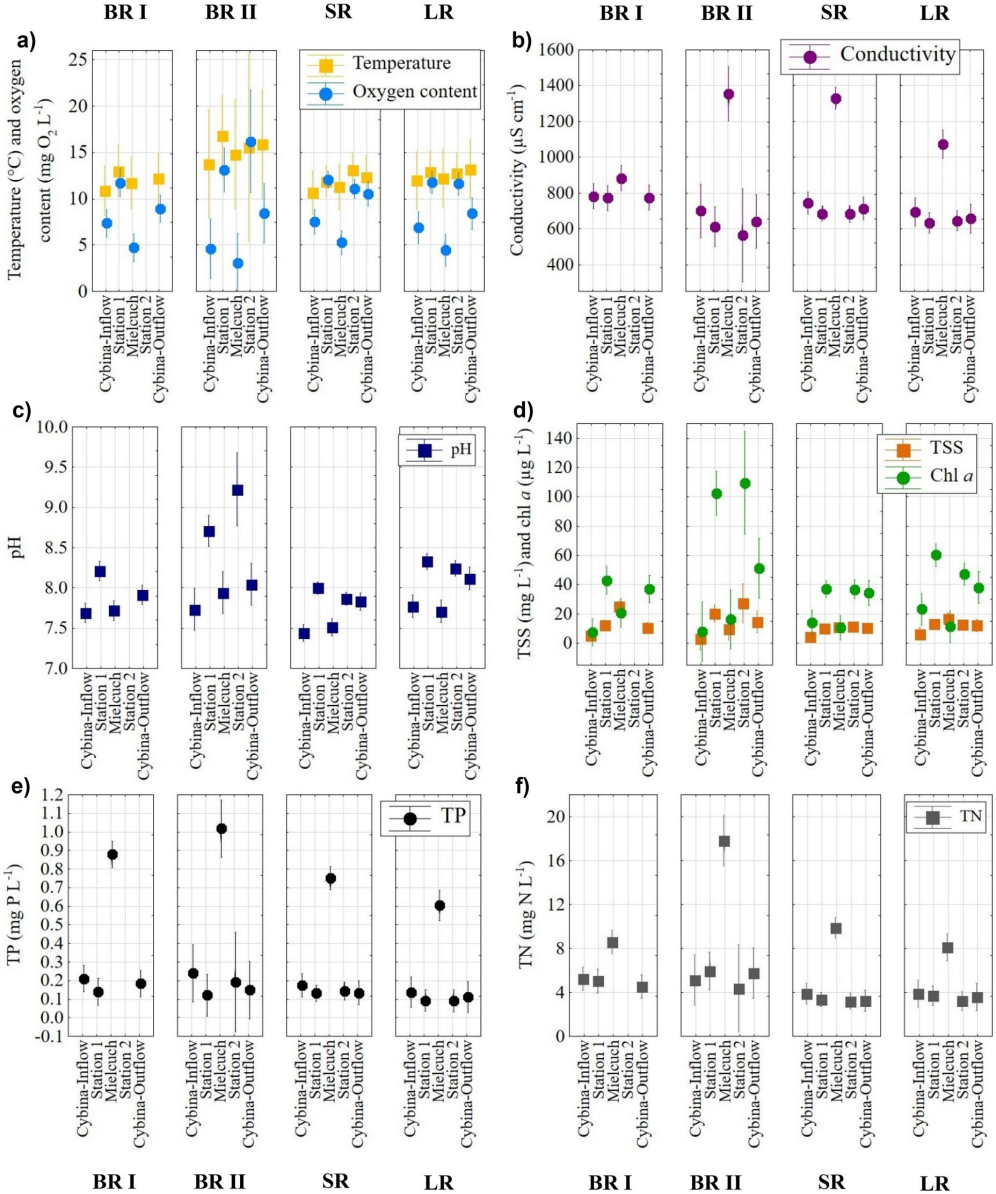
The mean (dot or square) with 0.95 confidence interval (whisker) of temperature and oxygen content (**a**), conductivity (**b**), pH (**c**), TSS content and chlorophyll *a* concentration (**d**), TP concentration (**e**) and TN concentration (**f**) during the 4 periods: BR I, BR II, SR and LR at 5 sampling stations: Cybina Inflow, Station 1, Mielcuch, Station 2, Cybina Outflow

The highest mean water temperature at all studied stations was noted during the BR II period (ca. 15°C). Regardless of the research period and restoration treatments, the water flowing through the lake warmed up. Thus, water at the outflow was about 0.5–2.0°C warmer in comparison with the inflows (Fig. 2a). There were no statistically significant differences between periods and stations (Table 2).

**Table 2:** Results of the two-way analysis of variance (ANOVA) for analysed parameters vs station and restoration period (bold parameters were significant)

	Station		Period		Station × period	
	*F*	*p*	*F*	*p*	*F*	*p*
Temperature	0.90	0.44	2.53	0.08	0.11	1.00
Conductivity	**93.33**	** < 0.01**	**9.79**	** < 0.01**	**9.24**	** < 0.01**
pH	**64.58**	** < 0.01**	**59.95**	** < 0.01**	**2.55**	** < 0.01**
Dissolved oxygen	**52.64**	** < 0.01**	1.12	0.33	1.15	0.32
TSS	**15.36**	** < 0.01**	**6.15**	** < 0.01**	**3.50**	** < 0.01**
Chl *a*	**60.34**	** < 0.01**	**18.33**	** < 0.01**	**4.71**	** < 0.01**
TN	**81.30**	** < 0.01**	**13.50**	** < 0.01**	**3.80**	** < 0.01**
TP	**182.94**	** < 0.01**	**6.80**	** < 0.01**	1.78	0.06

Higher oxygen content was recorded in the lake (Stations 1 and 2, 10.9–16.2 mg O_2_ L^−1^), especially during the BR II period, than in the river waters (3.1–10.3 mg O_2_ L^−1^). The dissolved oxygen content fluctuated at the Cybina Inflow at around 7.0 mg O_2_ L^−1^, reaching the highest values (7.6 ± 4.1 mg O_2_ L^−1^) during SR. The lowest dissolved oxygen concentration was noted in Mielcuch, where the oxygen content ranged from 3.1 ± 3.1 mg O_2_ L^−1^ in the BR II period to 5.3 ± 3.2 mg O_2_ L^−1^ during the SR period (Fig. 2a). The waters at the Cybina Outflow were better oxygenated than those in the tributaries but less than those in the lake. The dissolved oxygen concentration at all stations during the LR period was similar to the values recorded in the BR period. Changes between stations were statistically significant, mainly due to Mielcuch (Table 2).

The mean values of conductivity decreased over the years and stations. The highest conductivity values were recorded at the Mielcuch, especially during the BR II and SR periods (average above 1300 μS cm^−1^). Despite the high conductivity values on the tributaries, lower values were noted in the lake and at the Cybina Outflow, reaching in the LR period an average above 660 μS cm^−1^ (Fig. 2b). Changes between station, period and station × period were significant, mainly due to high values in Mielcuch (Fig. 2b, Table 2).

The pH of water in the studied rivers ranged from 6.3 to 9.2. The highest values were recorded in the lake, especially during the BR I, II and LR periods (average above 8.2), and the lowest during the SR period (Fig. 2c). The lowest values were observed at the Cybina Inflow and Mielcuch (average below 7.9), while at the Cybina Outflow, they were around 8.0. Differences between station, period and station × period were significant (Table 2).

Chl *a* concentration varied strongly depending on the station and the research period (Fig. 2d). The highest concentrations were recorded at Stations 1 and 2, where values ranged from above 100 μg L^−1^ (during BR II) to 40 μg L^−1^ (during SR). High results were also observed at the Cybina Outflow, where the values ranged from 34.5 ± 25.1 (during SR) to nearly 51.0 ± 28.0 μg L^−1^ (during BR II). An increase of chl *a* concentrations over the years was observed at the Cybina Inflow. In the BR period, values were low—on average, 7.5 ± 8.1 μg L^−1^—but afterwards they reached threefold higher values during the LR period—23.3 ± 28.9 μg L^−1^. Only in the Mielcuch was a twofold decrease of chl *a* concentration observed, from 16.2 ± 9.0 μg L^−1^ in the BR II period to 11.4 ± 10.7 μg L^−1^ during the LR period. Differences between station, period and station × period were significant (Table 2).

Total suspended solids (TSS) content varied between stations, especially during BR I and II, while values were more constant during the SR and LR periods (Fig. 2d). The lowest average concentrations of TSS, ca. 3.0 mg L^−1^, were observed at the Cybina Inflow (BR II), while those in the lake, at Stations 1 and 2, were the highest (18.8–27.5 mg L^−1^, BR II). The content of TSS in the Cybina Outflow was twofold higher than in the Cybina Inflow in all studied periods. In Mielcuch, the highest TSS values were recorded during the BR I period (26.9 ± 31.2 mg L^−1^), which decreased during the SR period (11.0 ± 7.7 mg L^−1^). The lowest TSS content was observed at all stations during the SR period. Differences between station, period and station × period were significant (Table 2).

**Fig. 3:**
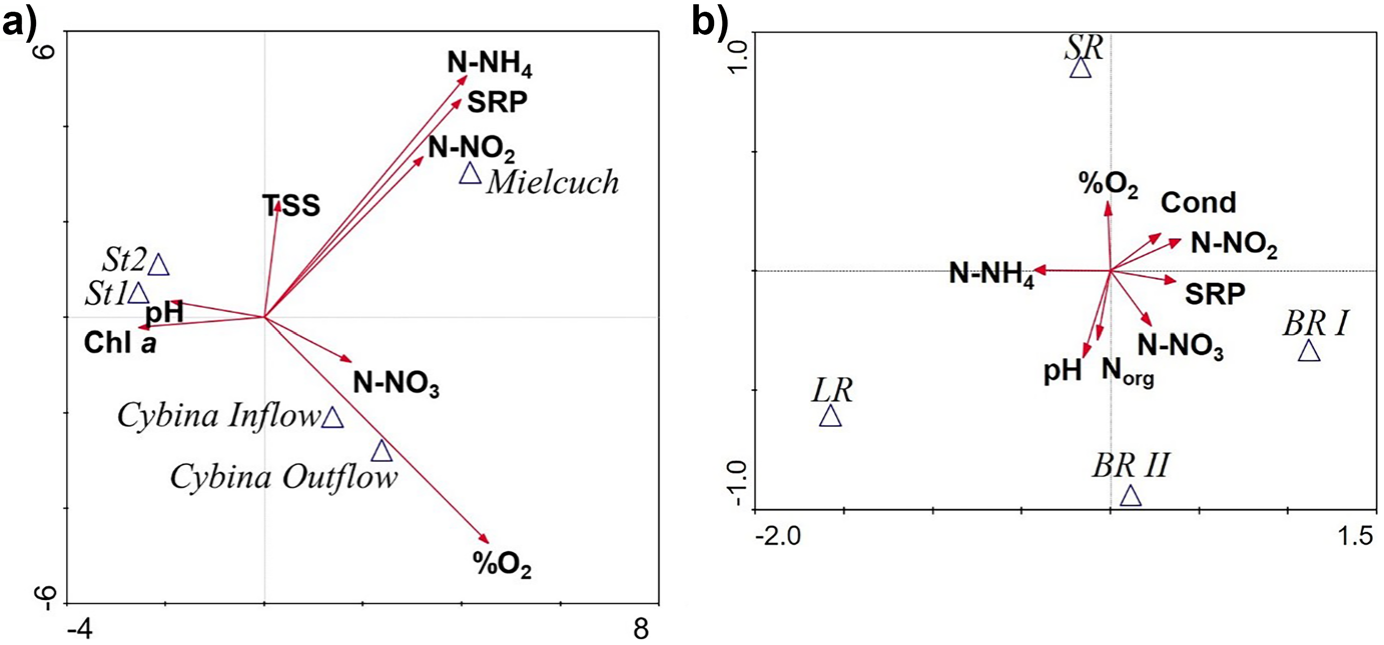
CVA diagrams of significant physico-chemical parameters depending on sampling stations (Cybina Inflow, Cybina Outflow, Mielcuch, St 1, Station 1; St 2, Station 2) during the whole studied period (**a**) and periods at the Cybina Outflow (BR I, 2000–2002; BR II, 2011; SR, 2012–2014; LR, 2015–2016) (**b**); the significance threshold *p* < 0.05

TP concentrations decreased over the years and at all stations from inflows to outflow (Fig. 2e). The highest concentration of TP was recorded during the BR I and II periods, especially in Mielcuch (even 1.01 ± 0.6 mg P L^−1^ in BR II). In addition, the mean TP concentration in Mielcuch was nearly fivefold higher than at the Cybina stations through the whole research period (Fig. 3e). The TP content was lower in the lake in comparison to that inflows and quite similar to the Cybina Outflow. There was a gradual decrease of phosphorus concentration during the restoration processes, especially in Mielcuch (up to 0.61 ± 0.40 mg P L^−1^) and at the Cybina Outflow (almost twofold, ca. 0.11 ± 0.03 mg P L^−1^). TP concentrations were also lower in the lake during restoration treatments (ca 0.09 mg P L^−1^). However, the values were still high. Differences between station and period were significant, mainly due to Mielcuch (Table 2). Nevertheless, the concentrations of TP at the outflow depended significantly on the quality of the tributaries, especially during the BR periods (Table 3; Spearman’s *ρ* > 0.69). TP concentrations in the lake also significantly impacted the outflow, mainly during restoration treatments (Table 3; Spearman’s *ρ* > 0.52). Regardless of the studied period, the main component of TP was phosphates (SRP).

**Table 3:** The results of the Spearman correlation (Spearman’s *ρ*) for TP and TN between outflow, lake stations and tributaries during studied periods (the significance threshold *p* < 0.05, bold results were significant)

**Station**		**Cybina Inflow**				**Station 1**				**Station 2**				**Mielcuch**			
**Period**		BR I	BR II	SR	LR	BR I	BR II	SR	LR	BR I	BR II	SR	LR	BR I	BR II	SR	LR
Cybina Outflow	TP	**0.69**	**0.83**	**0.40**	0.29	**0.69**	0.60	**0.64**	0.42	No data		**0.70**	**0.52**	**0.75**	**0.89**	0.27	0.24
	TN	**0.83**	1.00	**0.80**	**0.64**	**0.85**	**0.82**	**0.85**	**0.62**			**0.85**	**0.60**	**0.62**	–0.49	–0.01	0.08

The highest mean TN concentration was noted during the BR II periods, especially in Mielcuch (17.84 ± 8.7 mg N l^−1^), while at the other stations, the concentrations were three–fourfold lower (Fig. 2f). During the SR period, a decrease in the concentration of TN was observed at all stations, especially in the lake (by ca. 1.2–2.5 mg, reaching approx. 3.2 mg N L^−1^) and Mielcuch (reaching 9.79 ± 5.4 mg N L^−1^). During LR, the mean concentrations of TN at the Cybina Inflow and Outflow, as well as Stations 1 and 2, were similar and fluctuated at 3.2–3.9 mg N L^−1^, whereas in Mielcuch, it decreased to 8.1 ± 2.7 mg N L^−1^. The decreases of TN concentrations were associated with the reduction of mineral nitrogen forms (mainly ammonium nitrogen and nitrate nitrogen) and were observed at all stations when restoration treatments were carried out. Differences between station, period and station × period were significant, mainly due to Mielcuch (Tab. 2). TN concentrations at the outflow strongly depended on the quality of the Cybina Inflow and Stations 1 and 2 during the whole studied period (Table 3; Spearman’s *ρ* > 0.60). Mielcuch Stream also significantly impacted the outflow, but only during the BR I period (Spearman’s *ρ* = 0.62).

### The canonical variates analysis (CVA)

The results of the CVA indicated that the watercourses differed significantly in terms of nitrite and nitrate ion contents, SRP, TSS, pH, oxygen content and chl *a* (Fig. 3a, Table 4). Especially Mielcuch delivered high concentrations of mineral forms of nutrients. The longer the vector, the greater the significance for the diversity in the analysed set. The highest ammonium and nitrite ion contents and SRP were noted in Mielcuch, whereas in Cybina Outflow and Cybina Inflow, the concentration was lower (similar to those noted at Stations 1 and 2). At the lake (Stations 1 and 2), higher chl *a* and pH values were important factors, while well-oxygenated waters characterised the Cybina Outflow (Fig. 3a, Table 4). The overall percentage of explained variance was 53.2%.

**Table 4:** Results of Monte Carlo permutation test (in CVA) of sampling stations during the whole studied period (a) and of periods in the Cybina Outflow; values of *p* and *F* were calculated using the test with 999 permutations; the significance threshold *p* < 0.05) (*ns*, not significant)

Variables	(a) Sampling stations during the whole studied period		(b) Periods at the Cybina Outflow	
	*p*	*F*	*p*	*F*
%O_2_	0.002	130.21	0.002	9.84
N-NH_4_	0.002	147.23	0.002	6.56
Chl *a*	0.002	22.67	ns	
N-NO_2_	0.002	18.64	0.002	8.47
N-NO_3_	0.002	9.99	0.020	3.45
TSS	0.002	8.69	ns	
SRP	0.002	9.07	0.002	7.50
pH	0.038	2.57	0.018	3.87
Norg	ns		0.032	3.17
Cond	ns		0.018	3.85

Analysing the Cybina Outflow during different periods, it can be noticed that high concentrations of nutrients were recorded before restoration (nitrite nitrogen and phosphates in the BR I, organic nitrogen in BR II and nitrate nitrogen in both periods). The waters were well oxygenated during the SR period. Also, nutrient concentrations clearly decreased. However, during both sustainable and limited restoration, ammonium nitrogen was still an important factor (Fig. 3b, Table 4). The overall percentage of explained variance was 39.7%.

## Discussion

Both the quality of the inflows and the restoration treatments applied in the lake had a significant impact on the quality of the outflow. This is crucial for the ecological functioning and societal value of water ecosystems (Ekvall et al., 2014). Comparing periods before and during restoration treatments, water quality was gradually improved in the tributaries (Cybina Inflow and Mielcuch Stream) and Swarzędzkie Lake (Stations 1 and 2). This was connected with the regulation of water and sewage management within the catchment area (Kowalczewska-Madura & Gołdyn, 2006; Rosińska et al., 2018). The reduction of fertilisers used in agriculture in the lake catchment also led to a decrease in the nutrient contents at the inflows over the years. Nevertheless, high conductivity (above 1000 μS cm^−1^) and nutrient concentrations (TP and TN) in Mielcuch evidenced pollution, e.g. some illegal sewage discharges and surface runoff. Nutrient control in tributaries causes a time lag in the regeneration of ecosystems, contributing to a delayed water quality improvement at the outflow. This is mainly associated with internal nutrient loading from bottom sediments, which increases to compensate the reduced external loading (Sas, 1990; Søndergaard et al., 2007). Even so, the concentration of nitrogen and phosphorus at the outflow gradually decreased during the restoration processes of the Swarzędzkie Lake, which indicates the importance of the treatments in overcoming the resilience of the ecosystem. Improvement was associated with both the reduction of nutrient concentrations in the water column and the decrease of internal supply from bottom sediments (Kowalczewska-Madura et al., 2019). Lower concentrations of TP and TN were observed at Stations 1 and 2 and the Cybina Outflow during the SR and LR periods compared to tributaries during the BR I and BR II periods. As a result of the applied treatments (mainly inactivation of phosphorus using chemical substances), the nutrients were precipitated from the water column. In addition, the sorption capacity of the bottom sediments increased. Thus, less phosphorus was released from sediments. However, during the LR period when phosphorus inactivation was suspended, no phosphorus retention was observed (Kowalczewska-Madura et al., 2019). Although TP concentrations decreased about twofold during restoration processes, the values were still too high (ca. 0.1 mg P L^−1^) to observe a constant improvement of water quality. Significant changes in shallow lakes are observed when TP is reduced below 0.05–0.10 mg P L^−1^ (Jeppesen et al., 2007). Thus, treatments in Swarzędzkie Lake should not be limited to only one method because aeration seems to be insufficient.

Although high concentrations of TN reached the lake with the tributaries, the concentrations in the lake decreased from 6.5 mg N L^−1^ during the BR periods to 3.4–3.7 mg N L^−1^ during the restoration period. Lakes located in the course of a river decrease the concentration of transported nitrogen. This is due to the presence of many sites for effective denitrification (anaerobic organic sediments, vegetation), the formation of reserves of organic matter (Hillbricht-Ilkowska, 1999; Saunders & Kalff, 2001), and the assimilation of nitrates by primary producers (Chittoor Viswanathan et al., 2015). Swarzędzkie Lake has a wide vegetation belt and the presence of anaerobic bottom sediments creates sites for effective nitrate assimilation and denitrification (Kowalczewska-Madura & Gołdyn, 2009). The phytolittoral was particularly abundant at the inflow and outflow of Swarzędzkie Lake, and thus in key sites for the metabolism of nutrients (Hillbricht-Ilkowska, 1999; O’Hare et al., 2018; Piotrowicz et al., 2006). Dense communities of *Phragmitetum communis*, *Typhetum angustifoliae* and *Nupharo-Nymphaeetum albae* occupying a wide and shallow inflow area as well as *Ceratophylletum demersi* and *Nupharo-Nymphaeetum albae* densely overgrowing the shallow basin near the outflow (Rosińska et al., 2017) act as a “phyto-purification system”*.* As a result, they absorbed nutrients, which significantly supported the restoration treatments in Swarzędzkie Lake.

A transformation of suspension from low-organic to rich-organic with many planktonic organisms (Hillbricht-Ilkowska, 1999) was observed between the inflows and the outflow of Swarzędzkie Lake. The concentrations of chl *a* and total suspended solids content were closely correlated at the outflow, and their values were even two–threefold higher than those at the inflows. However, the values were lower than in the lake, where the results were the highest, especially in the BR II period. During that time, the intensive cyanobacterial bloom was observed (Kozak et al., 2014), that’s why the chl *a* and pH values were so high in that period (mean values above 100 g μL^−1^ and 8.6, respectively). It is likely that this was connected with higher temperature and high TP concentration at the Cybina Inflow and Mielcuch (ca. 0.25 mg P L^−1^ and 1.0 mg P L^−1^, respectively; Fig. 2a and e). The concentration of chl *a* at the Cybina Outflow was slightly lower during SR. Thus, restoration treatments and the presence of aquatic plants brought positive effects. The higher oxygen content in the lake and at the Cybina Outflow was related to the higher abundance of phytoplankton, reflected by the higher concentration of chl *a* and intensive photosynthesis (Chittoor Viswanathan et al., 2015). The lack of a relationship between chl *a* and nutrient concentrations was a result of coagulant application. The use of chemicals in lake restoration to remove phosphates is connected with strong changes in the physical and chemical features of water, leading to whole ecosystem disturbances (Rybak et al., 2020a, 2020b). Using chemicals is associated with the occurrence of a high amount of flocks, which absorb phytoplankton on their surface and transfer them to bottom sediments. Hence, during the SR period, the values of TSS (which consisted of phytoplankton) were the lowest and part of the matter was accumulated in the bottom. The outflow of nutrients from the lake, as well as suspended solids and chl *a*, was limited. Therefore, the water of better quality reaches downstream water bodies, and the lake could play a role as a pollution sink. However, TN concentration, chl *a* content and TSS values slightly increased during LR. Thus, changes in the application of the restoration methods (e.g. limiting some treatments) must be done carefully because the previously achieved effect may be lost.

The importance of restoration was also confirmed by the lack of a significant correlation between the concentration of nutrients in Mielcuch and the Cybina Outflow during restoration. However, earlier, this relationship was statistically significant (Table 3). Nevertheless, the impact of the Cybina Inflow and lake (Stations 1 and 2) was still important, including during the restoration processes.

## Conclusions

Both the quality of the inflows and the restoration treatments applied in the lake had a significant impact on the quality of the outflow. This is crucial for the ecological and societal value of the river–lake system as units for ecosystem services. Although in situ restoration processes do not affect the water quality in the inflows, nutrient control in the catchment area (regulation of water and wastewater management and reduction of fertilisers used in agriculture) is essential in obtaining a more effective improvement of the water quality in the through-flow lake and its outflow. However, the tributaries of Swarzędzkie Lake remained an important source of nutrients. In spite of this, lake restoration treatments enhanced the reduction of nutrient concentrations at the outflow of Swarzędzkie Lake compared to the period before restoration. Water at the outflow was also better oxygenated and with lower conductivity than at the inflows. However, higher contents of chl *a* and suspended solids were transported to the river below the lake, compared to the tributaries. Nevertheless, during the sustainable restoration period, their concentrations at the Cybina Outflow were lower than those before restoration and during limited restoration. Despite the limitation of restoration to one method, a decrease of total phosphorus was still observed, although the concentration of total nitrogen slightly increased. Sudden limitation of lake restoration may result in the loss of a previously achieved improvement and transform it into a source of pollution for the river and lakes below. During restoration treatments, it is important to focus on the water quality not only in the lake but also at the outflow. It must be remembered that in the case of cascade lakes, the outflow from the lake becomes a tributary to the next one. Hence, the restoration of the upstream lake and good quality of outflow are of great importance for all other lakes located downstream.

## Data Availability

The datasets used and/or analysed during the current study are available from the corresponding author on reasonable request.

## References

[CR1] Barałkiewicz D, Gramowska H, Kanecka A, Krzyżaniak I, Gołdyn R (2008). Spatial distribution of major and trace elements in the water of Swarzędzkie Lake (Poland). Environmental Monitoring and Assessment.

[CR2] Chittoor Viswanathan V, Molson J, Schirmer M (2015). Does river restoration affect diurnal and seasonal changes to surface water quality? A study along the Thur River, Switzerland. Science of the Total Environment.

[CR3] Directive. (2000). 2000/60/EC of the European Parliament and of the Council of 23 October 2000 establishing a framework for Community action in the field of water policy. OJL327/1 from 22.12.2000.

[CR4] Dondajewska R, Gołdyn R, Kowalczewska-Madura K, Kozak A, Romanowicz-Brzozowska W, Rosińska J, Budzyńska A, Podsiadłowski S, Korzeniewska E, Harnisz M (2020). Hypertrophic lakes and the results of their restoration in Western Poland. Polish river basins and lakes – part II.

[CR5] Dunalska JA, Napiórkowska-Krzebietke A, Ławniczak-Malińska A, Bogacka-Kapusta E, Wiśniewski G (2018). Restoration of flow-through lakes – theory and practice. Ecohydrology & Hydrobiology.

[CR6] Ekvall, M. K., Urrutia-Cordero, P., Hansson, L. -A. (2014). Linking cascading effects of fish predation and zooplankton grazing to reduced cyanobacterial biomass and toxin levels following biomanipulation. *PLoS One,**9*(11), e112956. 10.1371/journal.pone.0112956 PMC4237340 25409309

[CR7] Elbanowska, H., Zerbe, J., & Siepak, J. (1999). *Physicochemical water testing*, UAM University Press, Poznań, pp 231 (in Polish).

[CR8] Gołdyn, R., & Grabia, J. (1998). *Program ochrony wód rzeki Cybiny*. Urząd Miasta Poznania, Wydział Ochrony Środowiska, Poznań (in Polish).

[CR9] Gołdyn R, Podsiadłowski S, Dondajewska R, Kozak A (2014). The sustainable restoration of lakes—towards the challenges of the water framework directive. Ecohydrology & Hydrobiology.

[CR10] Gołdyn R, Szeląg-Wasielewska E (2004). Changes in the phytoseston of a river-lake system in Drawieński National Park. Oceanological and Hydrobiological Studies.

[CR11] Hatvani IG, Clement A, Korponai J, Kern Z, Kovács J (2017). Periodic signals of climatic variables and water quality in a river – eutrophic pond – wetland cascade ecosystem tracked by wavelet coherence analysis. Ecological Indicators.

[CR12] Hillbricht-Ilkowska A (1999). Shallow lakes in lowland river systems: Role in transport and transformations of nutrients and in biological diversity. Hydrobiologia.

[CR13] Jeppesen E, Meerhoff M, Jacobsen BA, Hansen RS, Søndergaard M, Jensen JP, Lauridsen TL, Mazzeo N, Branco CWC (2007). Restoration of shallow lakes by nutrient control and biomanipulation—the successful strategy varies with lake size and climate. Hydrobiologia.

[CR14] Klimaszyk P, Rzymski P, Piotrowicz R, Joniak T (2015). Contribution of surface runoff from forested areas to the chemistry of a through-flow lake. Environmental Earth Sciences.

[CR15] Kowalczewska-Madura K (2003). Mass balance calculations of nitrogen and phosphorus for Swarzędzkie Lake. Limnological Review.

[CR16] Kowalczewska-Madura, K. (2005). *Wpływ zmian obciążenia związkami biogennymi na strukturę i funkcjonowanie ekosystemu Jeziora Swarzędzkiego*. PhD thesis. Poznań.

[CR17] Kowalczewska-Madura K, Dondajewska R, Gołdyn R, Rosińska J, Podsiadłowski S (2019). Internal phosphorus loading as the response to complete and then limited sustainable restoration of a shallow lake. Annales De Limnologie - International Journal of Limnology.

[CR18] Kowalczewska-Madura K, Gołdyn R (2006). Anthropogenic changes in water quality in the Swarzędzkie Lake (West Poland). Limnological Review.

[CR19] Kowalczewska-Madura K, Gołdyn R (2009). Internal loading of phosphorus from sediments of Swarzędzkie Lake (Western Poland). Polish Journal of Environmental Studies.

[CR20] Kowalczewska-Madura, K., Gołdyn, R., & Lisowska, M. (2005). Seasonal changes in water quality of the Cybina River. In H. Gurgul (Ed.), *Physicochemical problems of natural waters ecology*. Vol. III. Szczecin, pp. 239–253 (in Polish).

[CR21] Kowalczewska-Madura K, Rosińska J, Dondajewska-Pielka R, Gołdyn R, Kaczmarek L (2020). The effects of limiting restoration treatments in a shallow urban lake. Water (switzerland).

[CR22] Kozak A, Kowalczewska-Madura K, Gołdyn R, Czart A (2014). Phytoplankton composition and physicochemical properties in Lake Swarzędzkie (midwestern Poland) during restoration: Preliminary result. Archives of Polish Fisheries.

[CR23] Kozak A, Rosińska J, Gołdyn R (2018). Changes in phytoplankton structure due to prematurely limited restoration treatments. Polish Journal of Environmental Studies.

[CR24] Krepski T, Czerniawski R (2018). Shaping of macroinvertebrate structures in a small fishless lowland stream exposed to anthropopressure, including the environmental conditions. Knowledge and Management of Aquatic Ecosystems.

[CR25] Kufel I, Kufel L (1997). Eutrophication processes in a shallow, macrophyte-dominated lake – nutrient loading to and flow through Lake Łuknajno (Poland). Hydrobiologia.

[CR26] Kufel L (1993). Particulate phosphorus sedimentation at the river inflow to a lake. Hydrobiologia.

[CR27] O’Hare MT, Aguiar FC, Asaeda T, Bakker ES, Chambers PA, Clayton JS, Elger A, Ferreira TM, Gross EM, Gunn IDM, Gurnell AM, Hellsten S, Hofstra DE, Li W, Mohr S, Puijalon S, Szoszkiewicz K, Willby NJ, Wood KA (2018). Plants in aquatic ecosystems: Current trends and future directions. Hydrobiologia.

[CR28] Piotrowicz R, Kraska M, Klimaszyk P, Szyper H, Joniak T (2006). Vegetation richness and nutrient loads in 16 lakes of Drawieński National Park (Northern Poland). Polish Journal of Environmental Studies.

[CR29] Precipitation data (Institute of Meteorology and Water Management) https://klimat.imgw.pl/pl. Accessed 29 January 2021.

[CR30] Pytka, A., Jóźwiakowski, K., Marzec, M., Gizińska, M., & Sosnowska, B. (2013). Impact assessment of anthropogenic pollution on water quality of Bochotniczanka River. *Infrastructure and Ecology of Rural Areas**3*(II), 15–29.

[CR31] Report. (2003). *Report on the state of the environment in the Wielkopolska Region in 2002*. The Voivodeship Inspectorate of Environmental Protection in Poznań, Library of Environmental Monitoring, Poznań (in Polish).

[CR32] Rosińska J, Gołdyn R (2015). Changes in macrophyte communities in Lake Swarzędzkie after the first year of restoration. Archives of Polish Fisheries.

[CR33] Rosińska J, Kozak A, Dondajewska R, Kowalczewska-Madura K, Gołdyn R (2018). Water quality response to sustainable restoration measures – case study of urban Swarzędzkie Lake. Ecological Indicators.

[CR34] Rosińska J, Romanowicz-Brzozowska W, Kozak A, Gołdyn R (2019). Zooplankton changes during bottom-up and top-down control due to sustainable restoration in a shallow urban lake. Environmental Science and Pollution Research.

[CR35] Rosińska J, Rybak M, Gołdyn R (2017). Patterns of macrophyte community recovery as a result of the restoration of a shallow urban lake. Aquatic Botany.

[CR36] Rybak M, Gąbka M, Ratajczak I, Woźniak M, Sobczyński T, Joniak T (2020). *In-situ* behavioural response and ecological stoichiometry adjustment of macroalgae (Characeae, Charophyceae) to iron overload: Implications for lake restoration. Water Research.

[CR37] Rybak M, Drzewiecka K, Woźniak M, Ratajczak I, Joniak T (2020). Iron-induced behavioural and biochemical responses of charophytes in consequence of phosphates coagulant addition: Threats to lake ecosystems restoration. Chemosphere.

[CR38] Sas H (1990). Lake restoration by reduction of nutrient loading: Expectations, experiences, extrapolations. Internationale Vereinigung Für Theoretische Und Angewandte Limnologie: Verhandlungen.

[CR39] Saunders DL, Kalff J (2001). Nitrogen retention in wetlands, lakes and rivers. Hydrobiologia.

[CR40] Søndergaard M, Jeppesen E, Lauridsen TL, Skov C, Van Nes EH, Roijackers R, Lammens E, Portielje R (2007). Lake restoration: Successes, failures and long-term effects. Journal of Applied Ecology.

[CR41] Stanford JA, Hauer R, Ward JV (1988). Serial discontinuity in a large river system. Internationale Vereinigung Für Theoretische Und Angewandte Limnologie: Verhandlungen.

[CR42] Szyper, H., Gołdyn, R., & Romanowicz, W. (1994). Lake Swarzędzkie and its influence upon the water quality of the River Cybina. In R. Gołdyn (Ed.), *Protection of the water of the catchment area of the River Cybina*, Prace Komisji Biologicznej PTPN 74, pp. 7–31.

[CR43] Tekile A, Kim I, Kim J (2015). Mini-review on river eutrophication and bottom improvement techniques, with special emphasis on the Nakdong River. Journal of Environmental Sciences.

[CR44] Teodoru C, Wehrli B (2005). Retention of sediments and nutrients in the Iron Gate I Reservoir on the Danube River. Biogeochemistry.

[CR45] Ter Braak, C. J. F., & Šmilauer, P. (2002). *CANOCO reference manual and CanoDraw for Windows user’s guide: Software for Canonical Community Ordination (version 4.5)*. Biometris, Wageningen.

[CR46] Tian Z, Zheng B, Wang L, Li H, Wang X (2017). Effects of river-lake interactions in water and sediment on phosphorus in Dongting Lake, China. Environmental Science and Pollution Research.

[CR47] Ward JV, Stanford JA, Fontaine TD, Bartell SM (1983). The serial discontinuity concept of lotic ecosystems. Dynamics of lotic ecosystems.

[CR48] Wetzel, R. G. (2001). *Limnology. Lake and river ecosystems*. 3rd Edition. Academic Press, pp. 1006.

